# Differences in Lectin‐Like Oxidized Low‐Density Lipoprotein Receptor‐1 Expression and Chemotaxis Between Neutrophils in the Oral Cavity and Those in Whole Blood

**DOI:** 10.1002/iid3.70390

**Published:** 2026-03-18

**Authors:** Richard F. Kraus, Vanessa J. Stampfer, Konstantin J. Scholz, Michael A. Gruber, Fabian Cieplik

**Affiliations:** ^1^ Department of Anaesthesiology University Medical Centre Regensburg Regensburg Germany; ^2^ Department of Conservative Dentistry and Periodontology University Medical Centre Regensburg Regensburg Germany; ^3^ Department of Operative Dentistry and Periodontology, Centre for Dental Medicine, Medical Centre – University of Freiburg, Faculty of Medicine University of Freiburg Freiburg Germany

**Keywords:** chemotaxis, LOX‐1, neutrophils, oral immunity, scanning electron microscopy (SEM), surface epitopes

## Abstract

**Aims:**

The abundance of neutrophils (polymorphonuclear cells, PMNs) in the oral cavity both in health and disease suggests that PMNs play a significant role in maintaining oral immune equilibrium. This cross‐sectional, in vitro study characterizes the surface epitopes, oxidative burst, and migratory properties of oral (oPMNs) and circulating (cPMNs) neutrophils in healthy volunteers.

**Methods:**

cPMNs were isolated from whole blood samples using gravity sediment separation; oPMNs were obtained from filtrated saliva. Surface epitopes and oxidative burst were analyzed with flow cytometry. PMN surface morphology was visualized with scanning electron microscopy (SEM) with gold labeling. A µ‐slide chemotaxis assay was conducted.

**Results:**

oPMNs showed significantly higher LOX‐1 expression than cPMNs (~1000 times). In contrast to oPMNs, cPMNs could be stimulated with PMA or TNF‐α, resulting in a significant LOX‐1 increase. PMA induced a stronger increase of LOX‐1 than TNF‐α. Only stimulation with TNF‐α or PMA resulted in the upregulation of CD11b and CD66b and the downregulation of CD62L on cPMNs. SEM showed an abundance of CD66b and diverging surface morphologies between oPMNs and cPMNs. Unlike cPMNs, oPMNs did not show chemotactic migration along an fMLP‐gradient.

**Conclusion:**

Our study highlights the high expression of LOX‐1 on oPMNs associated with a lack of further stimulability and absent in vitro chemotactic migration, indicating that these cells are in a higher state of activation than cPMNs. These findings provide new insights into the phenotypic and functional characteristics of oral neutrophils.

## Summary

- Background
  PMNs play a key role in maintaining healthy homeostasis in the oral cavity. However, many aspects, especially the nature and alteration of PMN surfaces, remain to be fully elucidated.
- Added value of this study
  To the best of our knowledge, this is the first study to document a naturally elevated expression of LOX‐1 on PMNs in the oral cavity. The different expression of the surface epitopes CD11b, CD66b, and CD62L additionally support a higher state of activation of oral PMNs. LOX‐1 appears to serve as a marker for PMNs that exhibit suppressive immune characteristics due to ongoing bacterial stimulation in the oral environment.
- Clinical implications
  This observation of surface changes, which was also visualized using scanning electron microscopy, improves our understanding of innate immunology in physiology. It also provides an essential basis for identifying changes in disease and potential therapeutic targets in pathological conditions (such as periodontitis).

## Graphical abstract

Polymorphonuclear neutrophils (PMNs) are crucial for oral health, yet their surface characteristics remain poorly understood. This study reveals elevated LOX‐1 expression on oral PMNs, indicating a heightened activation state, as evidenced by altered CD11b, CD66b, and CD62L levels. Scanning electron microscope images (as shown in this graphical abstract picture) illustrate this difference between PMNs from blood and oral cavity purely optical. These findings enhance our understanding of innate immunity and may inform future therapeutic strategies for conditions like periodontitis.

**Abbreviations**
cPMNs: circulating PMNs
fMLP: *N*‐formylmethionine‐leucyl‐phenylalanine
LOX‐1: lectin‐like oxidized low‐density lipoprotein receptor‐1
MFI: median fluorescence intensity
MPO: myeloperoxidase
NET: neutrophil extracellular trap
oPMNs: oral PMNs
oxLDL: oxidized low‐density lipoprotein
PMN: polymorphonuclear cell
ROS: reactive oxygen species
SEM: scanning electron microscope

## 1 Introduction

Once seen as merely antimicrobial effector cells equipped with cytotoxic substances, neutrophils (polymorphonuclear cells, PMNs) are now recognized as a key component in tissue inflammation. They are associated with a variety of chronic disorders, including cardiovascular and pulmonary diseases, sepsis or periodontitis. In cases of chronic inflammation, the immune mechanisms of PMN cells have the potential to injure healthy tissue. Thus, balanced PMN activity is indisputable for healthy tissue homeostasis [1, 2].

PMNs are produced in the bone marrow and released into the bloodstream. To reach the target tissues (such as the oral cavity), recruitment of PMN out of the bloodstream requires adhesion to and subsequent transmigration through the walls of vascular capillaries (extravasation) [2, 3]. The first step in PMN extravasation is the activation and upregulation of adhesion molecules. CD62L, one of the first PMN adhesion molecules to interact with the endothelium in the bloodstream, is crucial for transmigration through blood vessels. CD11b upregulation enables activated PMNs to adhere to the vascular endothelium, initiating transmigration [2, 4]. PMNs are commonly found in the oral cavity of healthy and diseased individuals. While most oPMNs migrate to the oral cavity through the gingival crevices, a small portion originate from the salivary glands. PMNs exit the gingival blood supply surrounding the teeth and then travel to the sulcus bottom. Subsequently, PMNs combine with the gingival crevicular fluid, which is constantly flowing into the oral cavity [5, 6]. About 30,000 oral PMNs per minute enter the oral cavity to maintain equilibrium [5, 6, 7, 8]. The movement of PMNs into the oral cavity is a continuous process and is found to be higher during the day and lower at night; however, the reason for the increase in the number of PMNs during the daytime is not known [5, 9].

PMNs–along with epithelial cells–are the predominant cells in the saliva [10]. Patients with chronic inflammatory conditions (such as in periodontitis and gingivitis) exhibit higher PMNs counts in the oral cavity than healthy persons [11, 12]. Such activation of PMNs in the oral cavity increases the level of CD66b (a PMN‐specific receptor with the main function to mediate phagocytosis) [5, 13]. Bactericidal enzymes as well as PMN‐released reactive oxygen species (ROS) eventually leak into the extracellular milieu, resulting in irreversible tissue destruction [4, 5]. As indicated in recent studies, the lectin‐like oxidized low‐density lipoprotein‐receptor‐1 (LOX‐1) is suggested to play a vital role in regulating such immunological processes involved in tissue homeostasis [14]. Originally described as receptor for oxidized low‐density lipoprotein (oxLDL), LOX‐1 was identified as an adhesion molecule for PMNs and is now recognized as a multiligand receptor that plays a role in a variety of different cellular processes [14, 15].

LOX‐1 is as a scavenger receptor that is primarily associated with endothelial dysfunction. It promotes this condition by inducing proatherogenic signaling and plaque formation in endothelial cells (ECs), via the uptake of oxLDL, which is a common byproduct of oxidative stress, and electronegative LDL [14]. The binding of oxLDL to LOX‐1 activates the receptor, resulting in the endocytosis of oxLDL by various cell types, such as macrophages, lymphocytes and PMNs, beyond ECs. This distribution suggests that the receptor may play a significant role in various aspects of inflammatory processes, in addition to its involvement in atherosclerotic plaque formation [14, 16, 17]. In macrophages, oxLDL uptake results in the formation of foam cells, which are a major component of atherosclerotic plaques. In addition to promoting oxLDL uptake, LOX‐1 triggers several signaling pathways, particularly the activation of the NF‐κB pathway, which plays a key role in inflammatory response. This activation increases the transcription of pro‐inflammatory cytokines, such as TNF‐α and IL‐6, and chemokines, such as MCP‐1, which then amplify inflammation and facilitate the recruitment of immune cells, including PMNs, to the sites of injury. Persistent uptake of oxLDL results in the generation of ROS, which causes oxidative stress and further cellular damage, aggravating inflammation [14].

PMNs from chronically inflamed tissue display a unique set of surface markers compared to tissue PMNs present in healthy, noninflamed tissue [13]. However, this has not yet been investigated in detail for oPMNs.

LOX‐1 has been implicated in modulating neutrophil activation and adhesion. Recent evidence suggests that its engagement can alter cytoskeletal dynamics and cell motility, thereby potentially influencing chemotactic behavior. Therefore, we hypothesized that oPMNs are in a state of higher activation than circulating PMNs (cPMNs), as evidenced by LOX‐1 upregulation and altered migratory properties.

Accordingly, to further investigate the activation status of PMNs in different environments, the aim of this study was to characterize surface epitopes (CD11b, CD62L, CD66b, and LOX‐1), oxidative burst, and migratory properties of oPMNs and cPMNs in healthy volunteers.

## 2 Methods

PMN samples of saliva and peripheral blood were analyzed for expression of the surface markers CD62L, CD11b, CD66b, and LOX‐1 using flow cytometry assays. The chemotactic properties of oPMNs and cPMNs were investigated with an established µ‐slide chemotaxis assay [3, 18, 19]; scanning electron microscopy (SEM) was used to visualize the PMN surface structure in detail.

### 2.1 Ethical Considerations

This study was approved by the Internal Review Board of the University of Regensburg (Reference 22‐3062‐101). Written informed consent was obtained from all volunteers included in this study before blood sampling. No external funding was received.

### 2.2 Sample Collection and Preparation

Peripheral blood PMNs (cPMNs) were isolated from the blood of healthy, Caucasian, nonsmoking donors, aged 20–30 years (*n* = 14, 10 women, 4 men), without gingivitis, periodontitis, or oral cancer, as described previously in detail by Hundhammer et al. [18]. All participants demonstrated good oral hygiene and were diagnosed as periodontally healthy patients. None of them had conditions or infections affecting PMNs, and all provided written informed consent. Furthermore, our healthy volunteers were free of chronic or recent acute illnesses (no infections within the preceding 4 weeks) and did not use any immunomodulatory medications or hormonal treatments (e.g., oral contraceptives or hormone replacement therapy). All vaccinations had been administered at least 4 weeks prior to sampling. To minimize dietary and lifestyle confounders, subjects abstained from dietary supplements, alcohol consumption, and vigorous physical activity for 12 h before sample collection. Blood was collected between 08:00 and 10:00 a.m. to control for circadian fluctuations in PMN counts.

Blood was collected using one lithium‐heparin‐anticoagulated tube and one serum clot activator tube (all blood collection materials were from SARSTEDT AG & Co. KG, Nuembrecht, Germany). PMNs were isolated without centrifugation. Therefore, 10% gelafundin ISO (40 mg/mL; B. Braun SE, Melsungen, Germany) was added to the whole blood. The supernatant containing most native PMNs was collected after 30 min [18]. To isolate oPMNs, saliva samples were taken from the same healthy donors, collected in a 50 mL centrifuge tube and diluted 1:1 with PBS and then sieved through a 40 µm cell strainer to separate the mucins from the cells (see Supporting Information S1: Figure 1). Centrifugation was avoided throughout both isolation procedures [13, 20].

### 2.3 Cell Counts in Saliva

For the initial analysis, four samples of filtered saliva were analyzed by counting cells with the “CASY” cell counter system (Omni live science, Bremen, Germany). For this purpose, 20 µL of filtered saliva was pipetted into preprepared 5 mL “Casyton‐solution” (Omni live science, dilution 1:25) and mixed gently. For each measurement, 3 × 400 µL were aspirated from the CASY device, and a mean value (MV) of the cell number was calculated from three measurements per subject. The threshold to preferentially count PMNs was set between 7.7 and 12.0 µm.

### 2.4 Flow Cytometry

Surface antigen expression levels and oxidative burst were measured by flow cytometry with a FACSCalibur flow cytometer and CellQuest Pro software Version 5.2 (both BD Biosciences, Franklin Lakes, New Jersey, USA). The data generated by the flow cytometer were analyzed using FlowJo (Version 10.0.7 LLC, Ashland, Oregon, USA) [19]. Cell counts of 5000 cells per sample were investigated. The gating strategies are shown in Supporting Information S2–S4: Figures 2–4. The different surface epitope expressions as well as the different oxidative burst reactions were assessed by comparing the median fluorescence intensities (MFIs).

#### 2.4.1 Analysis of Surface Antigen Expression

For the surface epitope analysis, PMNs were identified by their typical patterns displayed in forward‐scatter (FSC) and side‐scatter (SSC) light and additionally by immunostaining with the PMN surface marker. CD15 (and also CD66b see below) were used as PMN surface markers to ensure that all cells analyzed were indeed of the neutrophil type. Living cells were further analyzed to assess the surface epitope expression [19].

For immunophenotyping, cell‐surface expression of various antigens on PMNs was investigated using the following commercially available antibodies, labeled either with phycoerythrin (PE), fluorescein isothiocyanate (FITC), or allophycocyanin (APC) according to the manufacturer's protocol [19]: one part of the samples was stained with APC‐conjugated antiCD66b (G10F5, BioLegend), PE‐conjugated antiCD11b (ICRF44, BioLegend), and FITC‐conjugated antiCD62L (DREG‐65, BioLegend). The other samples were stained with FITC‐conjugated antiCD15 (301904, BioLegend, San Diego, CA, USA) and APC‐conjugated antiLOX‐1 (130‐122‐111, MiltenyiBiotec, Bergisch Gladbach, Germany). The immunostaining process involved two centrifugation steps that were both performed at 4°C immediately before flow cytometric measurement [18]. We measured the samples sequentially, ensuring that CD66b and LOX‐1 were never analyzed simultaneously by flow cytometry, which allowed us to distinguish between them effectively.

Two different gating strategies were applied:

The first staining and gating strategy involved running the samples via FSC‐H + SSC‐H, followed by sorting of antiCD66b (G10F5, BioLegend) and concluding with the division into CD62L and CD11b [18].

The second strategy involved running the samples FSC‐H + SSC‐H, followed by sorting of FITC‐conjugated antiCD15 (301904, BioLegend, San Diego, CA, USA) and ultimately analyzing LOX‐1.

In addition, samples of leukocyte‐rich supernatant and saliva (see Section 2.2) were mixed with tumor necrosis factor alpha (TNF‐α; 1 µg/mL, Thermo Fisher Scientific, Waltham, Massachusetts, USA) and fMLP (10 µM, Sigma Aldrich, St. Louis, Missouri, USA) or phorbol‐12‐myristate‐13‐acetate (PMA; 10 µM, Sigma Aldrich). Additionally, surface epitope antibodies (antiCD66b‐APC, antiCD11b‐PE, and antiCD62L‐FITC or antiCD15‐FITC and antiLOX‐1‐APC; see above) were given to samples of leukocyte‐rich supernatant and saliva samples after 20, 60 and 120 min of stimulation with PMA and TNF‐α, to investigate the time component of activation.

Surface epitope analysis was performed in the same way for each antibody used and was applied to both cPMNs and oPMNs. The results were summarized as numerical value of the median of each point cloud and tabulated in Microsoft Excel (Microsoft Excel Version 2016, Microsoft Corporation, Redmond, Washington, USA).

#### 2.4.2 Analysis of Neutrophil Oxidative Burst

Oxidative burst was investigated by flow cytometry directly after PMN isolation. Samples were taken from the leukocyte‐rich supernatants gained in the isolation process and not subjected to any further treatment. Burst was stimulated by fMLP (10 µM, Sigma Aldrich) in combination with TNF‐α (1 µg/mL, Thermo Fisher Scientific) or with PMA (10 µM, Sigma Aldrich), which was set as a positive control [18]. ROS production was detected by oxidation of nonfluorescent Dihydrorhodamine (DHR) 123 (10 µM, Thermo Fisher Scientific) to fluorescent Rhodamine 123.

Analyses of the ROS experiments were also performed with FlowJo in the same way as the surface epitope experiments (see above). Once again, granulocytes were gated in SSC versus FSC. Dead cells were stained with 10 μL of propidium iodide (PI, 1.5 mM, Invitrogen, Thermo Fisher Scientific, Waltham, Massachusetts, USA). Only PI‐negative (living) cells were included in subsequent analyses to assess their ROS production, for which purpose Rhodamine 123 fluorescence was measured [4].

### 2.5 Chemotaxis Model

To measure the migratory abilities of cPMNs and oPMNs, we conducted a chemotaxis model as previously described by Hundhammer et al. [18]. Cells were analyzed by fluorescence light microscopy in 3D‐µ‐slide chemotaxis chambers (IBIDI GmbH, Martinsried, Germany). Each 3D‐µ‐slide consisted of three channels, which were filled with type I collagen gel (about 7 µL with 1.5 mg/mL PureCol, Advanced BioMatrix Inc., Carlsbad, California, USA). Each channel was flanked by two reservoirs (65 µL each); one side was filled with partially isolated (oral or circulating) PMNs and the other side with RPMI and a chemoattractant (10 nM fMLP, Sigma‐Aldrich) [3, 18, 19], which created a chemotaxis gradient in the collagen gel. Migration was measured using a DMi8 inversion microscope (Leica Microsystems CMS GmbH, Wetzlar, Germany). Phase‐contrast images were obtained using a DFC9000 GT SCMOS monochrome camera (Leica Microsystems) as described before [3]. The effectively covered PMN migration distance was measured as TrackLength [μm]. Control of the microscope and the observation process was computer‐aided using the Application Suite X software platform (Leica Microsystems) [3, 19].

### 2.6 Scanning Electron Microscopy

To prepare cPMNs for SEM, sample collection and PMN isolation as described above were performed for both oPMNs and cPMNs. cPMNs were taken directly from the supernatant of the cPMNs after sedimentation (for details, see Section 2.2), and cPMNs were collected after migrating through the collagen gel of the chemotaxis chamber under their own power (see Section 3.2.5, 5). The oPMNs were prepared for SEM in the same way with filtration steps as described above (see Section 2.2).

For some of the experiments, prepared PMNs were additionally conjugated with antibody‐coupled gold beads to CD66b using NanoComposix Gold (80 nm) Conjugation Kit for Lateral Flow (UZR80‐10MC, nanoComposix, San Diego, CA, USA). Anti‐CD66b (G10F5, BioLegend) was desalted according to the manufacturer's antibody desalting/buffer exchange protocol. The antibodies were then conjugated with the gold beads in several centrifugation (3000 rpm; 865 *g*) and washing steps with reaction buffer [21]. PMNs were carefully mixed with beads for 15 min. This procedure was performed for one specimen of cPMNs (migrated and unmigrated) and oPMNs each, as well as for the supernatant (cPMN) and the saliva alone (oPMN) to analyze potential false‐positive gold‐labeling results.

The prepared cells were placed into an insert chamber (tissue culture coverslip (#83.1840.002; Sarstedt) in a 24‐well culture plate. PMNs were allowed to settle on the round plates in the chamber. After 1 h, the supernatant was removed, and the filtrate was applied again. The cells were fixed in glutaraldehyde (2.5% in Sörensen buffer, pH 7.4) for 2 h and subsequently washed with Sörensen buffer. The buffer was then carefully removed [22], and the cells were subjected to an ascending series of alcohol concentrations (30%, 50%, and 70% ethanol), each for 10 min. Samples were stored in 70% ethanol for 24 h at 4°C. The next day, the ascending alcohol series was continued (series started with 80%, 90%, and 96% ethanol and ended with pure ethanol). The samples were finally dried in a critical point dryer (Leica EM CPD300, Leica Microsystems, Wetzlar, Germany) using ethanol absolute as the intermediate medium and CO_2_ as the drying medium.

The specimens were mounted onto aluminum stubs (Leit‐Plastcluste BP2080, 12.5 mm Ø BP 2152, BALTIC‐Präparation e.K., Wetter, Germany) using double‐sided adhesive carbon discs and conductive adhesive paste (Leit‐Plast BP2080, 12 mm Ø G 3347, BALTIC‐Präparation e.K.). The samples were then sputter‐coated with platinum for 50 s, 30 mA argon gas (Pt layer thickness of ~4–5 nm) using a sputter coater (BAL‐TEC SCD 005, BALTIC‐Präparation e. K.). The specimens were examined with an SEM (FEI Qanta 400 FEGI, FEI Company, Thermo Fisher Scientific, FEI Deutschland GmbH, Dreieich, Germany; high vacuum mode, backscatter electron mode, accelerating voltage 10 kV, spotsize 3, working distance 6–7 mm, aperture 30 µm ø) [22]. Additionally, exemplary EDX mappings were used to visualize elemental gold (high vacuum, 10 kV, original magnification ×10,000, resolution 256 × 200 pixels, filter time 1.92 µs; EDAX Octane Elect detector, APEX v2.5 AMETEK EDAX GmbH, Unterschleissheim, Germany).

### 2.7 Statistical Analysis

Statistical analysis was carried out using SPSS Statistics Version 29 (IBM) [19]. Normal distribution was verified by the Kolmogorov–Smirnov test. With normal distribution and in case of multiple comparisons and existing homogeneity of variance, the MV were checked for significant differences by a single‐factor analysis of variance (ANOVA) with indication of the standard deviation ± SD. If there was no homogeneity of variance, Welch's ANOVA test was applied. With existing homogeneity of variance, the subsequent post hoc analysis was done according to Bonferroni. In case of existing inhomogeneity of variance, Dunnet's T3 test was used. The test for homogeneity of variance was conducted using Levene's test. If there was no normal distribution in the data, the central tendencies of the individual groups were compared with Kruskal–Wallis followed by pairwise comparison (more than two groups with post hoc testing according to Dunn–Bonferroni) or Mann–Whitney *U* (two groups) testing, indicating the median and the interquartile range. Detailed information on used post hoc tests can be found in Supporting Information S5: Table 2. The distribution of results is visualized with bar graphs indicating mean ± 95% confidence interval or as boxplot (see Figure 3a) displaying median, lower quartile, and calculated minima and maxima. Statistical outliers are represented as circles, and extreme values are depicted as asterisks. An error probability of *p* < 0.05 was considered statistically significant [3].

## 3 Results

### 3.1 Cell Counts

The healthy participants exhibited a physiological concentration of PMNs in their blood (as reported here [23]).

In total, 14 healthy volunteers (10 women, 4 men) were enrolled. The mean age was 25.1 ± 3.2 years (range: 20–30 years), and the mean body mass index (BMI) was 22.7 ± 2.1 kg/m^2^. The counts of cPMNs and oPMNs did not differ significantly between women and men, indicating no sex‑related bias in our primary outcome. This demographic overview enhances the transparency and reproducibility of our findings.

The measurement of 20 µL of filtrated saliva (*n* = 4) showed a mean cell number (oPMNs) of 410,000 ± 80,700 cells/mL. After blood sedimentation, the mean concentration was ~800 ± 40 PMNs/µL (cPMNs) of extract. Thereby, an average of 29.8% ± 19.3% of the total number of cells measured by flow cytometry were identified as cPMNs.

### 3.2 Analysis of Surface Epitopes With Flow Cytometry

The following results were generated between 0 and 120 min incubation. After 120 min, no PMNs were detectable in the FACS upon PMA activation. The results were summarized when no further differences were found in the temporal course.

#### 3.2.1 Expression of Surface Epitopes on Oral and Circulating PMNs

##### 3.2.1.1 Expression of LOX‐1

Oral PMNs expressed significantly more LOX‐1 on their surface than cPMNs at time *t* = 0 min (*p* < 0.001), see Figure 1a). Regardless of the incubation time, oPMNs showed no significant differences of LOX‐1 expression upon stimulation with TNF‐α or PMA (*p* > 0.05) versus control. The MFI of LOX‐1 was at a high level in oPMNs at all time points (0–120 min) and differed significantly from the MFI of LOX‐1 of cPMNs at all time points (Figure 1b; no activation (*p* < 0.001); TNF‐α (*p* < 0.001); PMA (*p* < 0.001). At each time point (20, 40, and 60 min), PMA induced higher MFI of LOX‐1 than TNF‐α in cPMNs (*p* values, see Figure 1a). After 20‐min incubation with PMA (*p* = 0.05) or TNF‐α (*p* = 0.002), LOX‐1 expression of cPMNs was significantly higher than without stimulation (the results are summarized in Figure 1a).

**Figure 1:**
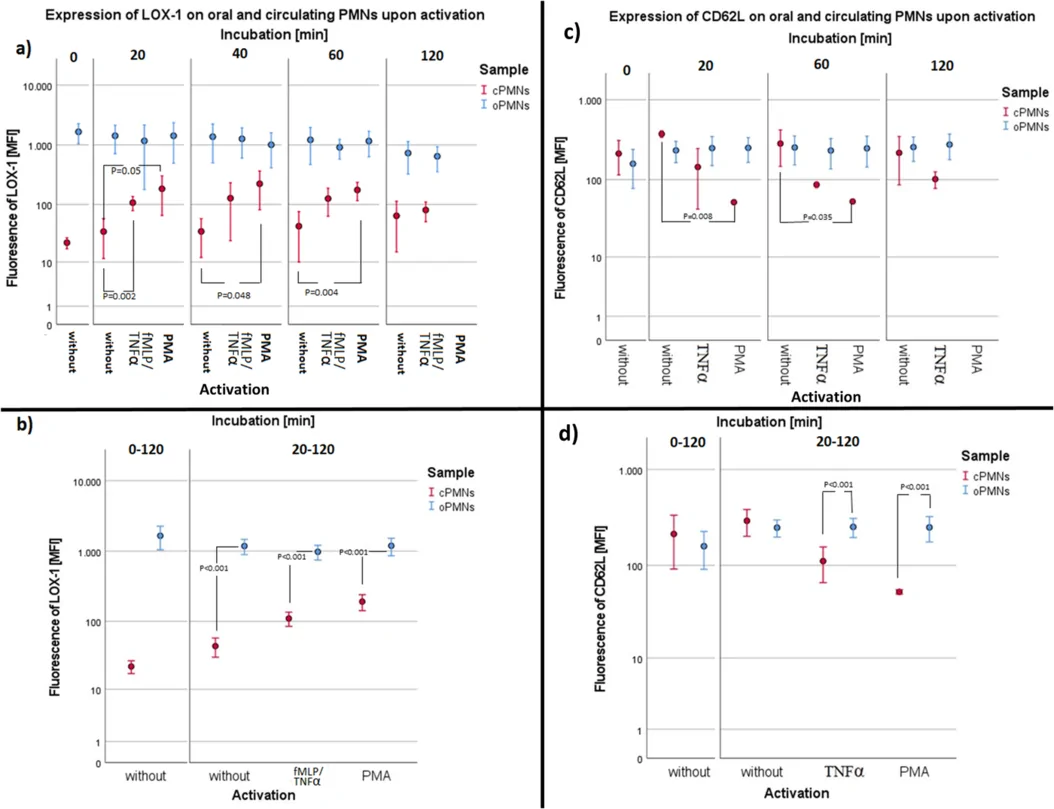
Comparison of LOX‐1 and CD62L expression of oral and circulating PMNs dependent on the activation with PMA or TNF‐α*. (a) The median fluorescence (MFI) of LOX‐1‐APC on oPMNs and cPMNs (*y*‐axis) is shown to be dependent on the activation with TNF‐α, PMA, or without activation (*n* = 14)^†‡^. (b) The *y*‐axis shows median fluorescence intensity (MFI) of LOX‐1‐APC, whereas the *x*‐axis distinguishes the activation of the PMN samples (*n* = 14)^‡^. (c) The median fluorescence (MFI) of CD62L‐FITC on oPMNs and cPMNs (*y*‐axis) is shown to be dependent on the activation with TNF‐α, PMA or without activation (*n* = 14)^†‡^. (d) The *y*‐axis shows median fluorescence intensity (MFI) of CD62L‐FITC, whereas the *x*‐axis distinguishes the activation of the PMN samples (*n* = 14)^‡^. *There were no significant differences between the incubation times of 20, 60, and 120 min; the values did not change significantly after 20‐min incubation. The results from 20 to 120 min incubation are summarized in (b) and (d) for a better overview. ^†^A distinction is made between oPMNs or cPMNs and by the incubation time of 0, 20, 60, or 120 min. ^‡^The cluster variable represents the origin of the samples.

##### 3.2.1.2 Expression of CD62L

cPMNs expressed CD62L at the same level as oPMNs at time *T* = 0 min and without stimulation (see Figure 1c,d). The expression of CD62L was not decreased on oPMNs by either TNF‐α or PMA (*p* > 0.05) compared to the nonactivated PMNs at the same time. On cPMNs, expression of CD62L was significantly decreased by PMA activation at 20 and 60 min (see Figure 1c,d).

##### 3.2.1.3 Expression of CD11b

The expression of CD11b was significantly increased on cPMNs compared to that on oPMNs at *t* = 0 min (*p* = 0.02); see Figure 2a,b). The expression of CD11b was significantly increased on oPMNs in all groups (without activation, with TNF‐α or with PMA; *p* < 0.001 each). CD11b showed no significant change after activation, neither on oPMNs nor on cPMNs (*p* > 0.05).

**Figure 2:**
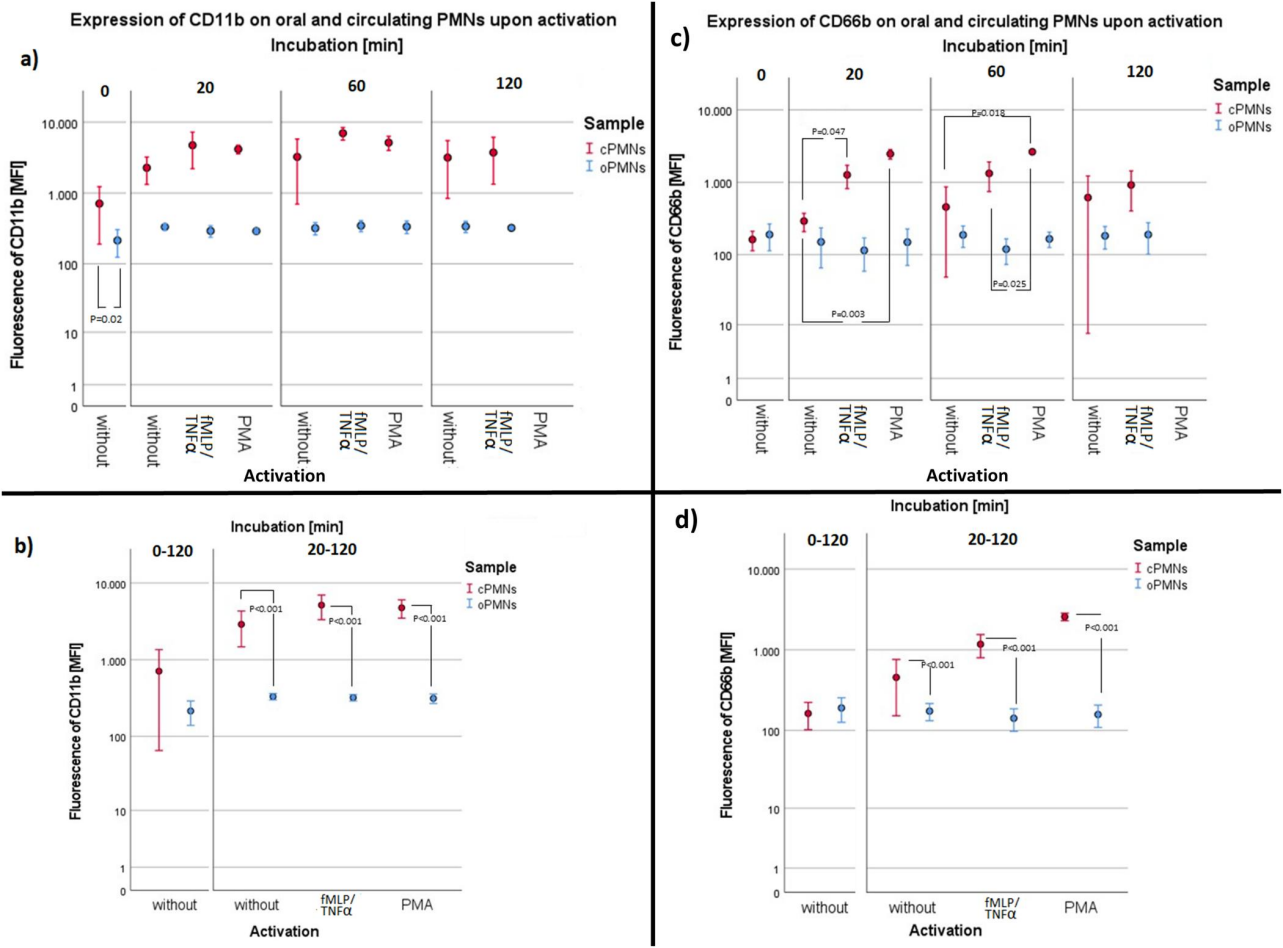
Comparison of CD11b and CD66b expression of oral and circulating PMNs dependent on the activation with PMA or TNF‐α*. (a) The median fluorescence (MFI) of CD11b‐PE on oPMNs and cPMNs (*y*‐axis) is shown to be dependent on the activation with TNF‐α, PMA or without activation (*n* = 14)^†‡^. (b) The *y*‐axis shows median fluorescence intensity (MFI) of CD11b‐PE, whereas the *x*‐axis distinguishes the activation of the PMN sample (*n* = 14)^‡^. (c) The *y*‐axis shows median fluorescence intensity (MFI) of CD66b‐APC, whereas the *x*‐axis distinguishes the activation of the PMN sample (*n* = 14)^‡^. (d) The median fluorescence (MFI) of CD66b‐APC on oPMNs and cPMNs (*y*‐axis) is shown to be dependent on the activation with TNF‐α, PMA, or without activation (*n* = 14)^†‡^. *There were no significant differences between the incubation times of 20, 60, and 120 min; the values did not change significantly after 20‐min incubation. The results from 20 to 120 min incubation are summarized in (b) and (d) for a better overview. ^†^A distinction is made between oPMNs or cPMNs and by the incubation time of 0, 20, 60 or 120 min. ^‡^The cluster variable represents the origin of the samples.

##### 3.2.1.4 Expression of CD66b

The expression of CD66b showed no significant differences, neither on oPMNs nor on cPMNs at *t* = 0 min (*p* > 0.05; see Figure 2c). The expression of CD66b was significantly increased after activation with TNF‐α, as well as PMA on cPMNs (*p* values see Figure 2c). When combining results beyond 20 min of incubation, cPMNs had a higher expression of CD66b (Figure 2d). CD66b on oPMNs showed no significant change after activation (*p* > 0.05).

#### 3.2.2 Oxidative Burst

Without stimulation, MFI of Rhodamine 123 of oPMNs was significantly higher than MFI of Rhodamine 123 of cPMNs (see Figure 3a). oPMNs showed no significant change in burst after activation (either fMLP/TNF‐α or PMA) (*p* > 0.05). cPMNs showed increased MFI of Rhodamine 123 when activated with fMLP/TNF‐α or PMA (see Figure 3a).

**Figure 3:**
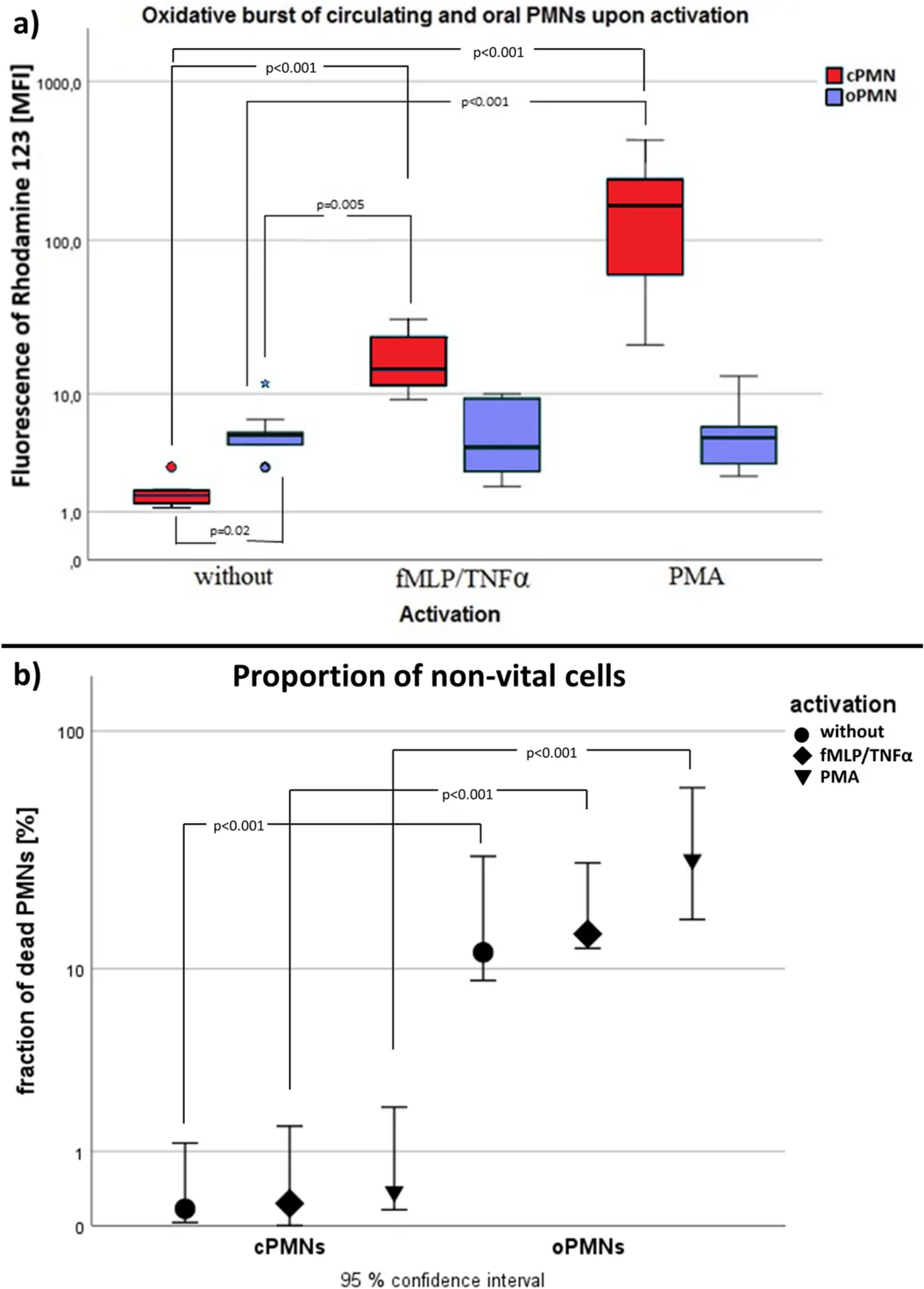
(a) Comparison of the oxidative burst of oral and circulating PMNs dependent on the activation with fMLP/TNF‐α or PMA. The *y*‐axis shows median fluorescence intensity (MFI) of DHR, whereas the *x*‐axis distinguishes the activation of the PMN sample (*n* = 14)^‡^. (b) Comparison of the proportion of nonvital cells subdivided into cPMN and oPMN dependent on the activation. The data are shown as median with a confidence interval (*n* = 14). ^‡^The cluster variable represents the origin of the samples.

#### 3.2.3 Cell Viability

The proportion of dead cells after stimulation differed between cPMNs and oPMNs (*p* < 0.001): it was < 1% in cPMNs, but had increased from 10% to 30% in oPMNs (see Figure 3b and Supporting Information S7: Table 1).

#### 3.2.4 Morphological Characterization Using Scanning Electron Microscopy

SEM revealed different surface properties of cPMN and oPMN. Directly after isolation by sedimentation, the surface of cPMNs was characterized by an abundance of microvilli‐like protrusions and constructs consistent with exocytosis particles. cPMNs that had migrated though collagen gel of our chemotaxis model (see Section 2.5) showed a smoother surface structure. oPMNs displayed a perforated structure near net‐like formations, which may be indicative of the activation and release of Neutrophil Extracellular Traps (NETosis, see Figure 4).

**Figure 4:**
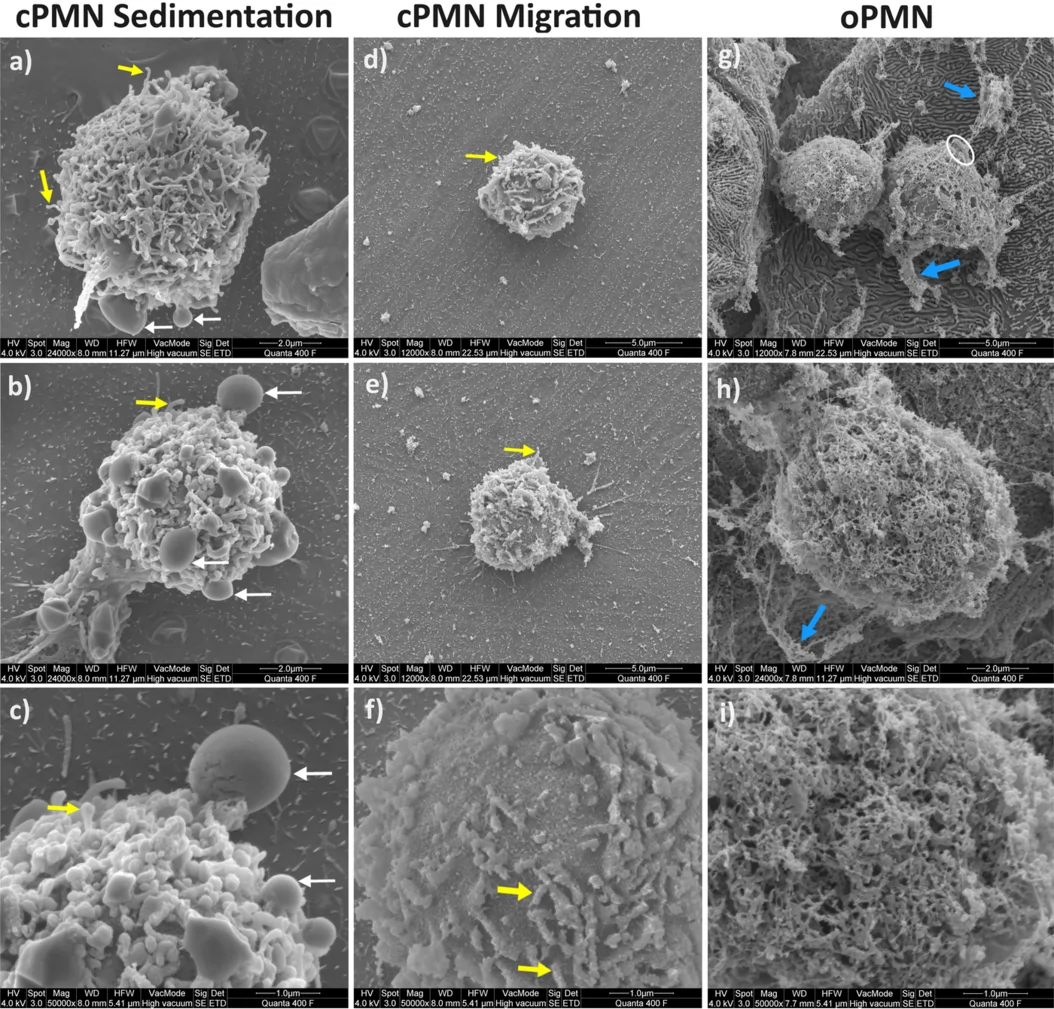
Cells compatible with polymorphic neutrophil granulocytes by morphology and size [24] visualized with a scanning electron microscope. PMNs showed different surface characteristics depending on the body region and preparation procedure. (a–c) cPMN from a blood sample after sedimentation with superficial morphology were consistent with granule exocytosis (white arrows) and microvilli‐like protrusions and membrane ruffles (yellow arrows) [25, 26]. (d–f) cPMN from a blood sample after migration through an extracellular collagen matrix. Significantly changed surface structure after migration [27]. Microvilli‐like protrusions and membrane ruffles (yellow arrows) appear to flatten and diminish in number [25, 28]. (g–i) oPMNs from a salivary sample. Perforated surface structure compatible with activated PMNs [24]. Mesh‐like NET structure (blue arrows) consisting of relatively uniforms fibers characteristic for NETosis [29, 30, 31]. Background: indicated biofilm with bacteria entrapped in nets (white ellipse) [32, 33, 34].

Labeling of the individual isolated cPMN samples with antibodies coupled to gold beads against CD66b revealed different distribution patterns on the surfaces of the PMNs. For example, we could show that CD66b is never distributed homogeneously on the PMNs' surface but it is located on a small number of specific microvilli (Figure 5‐1), which was reduced to one sole spot after migration (Figure 6). The presence of elemental gold was additionally confirmed by exemplary EDX mapping (see Figure 7).

**Figure 5:**
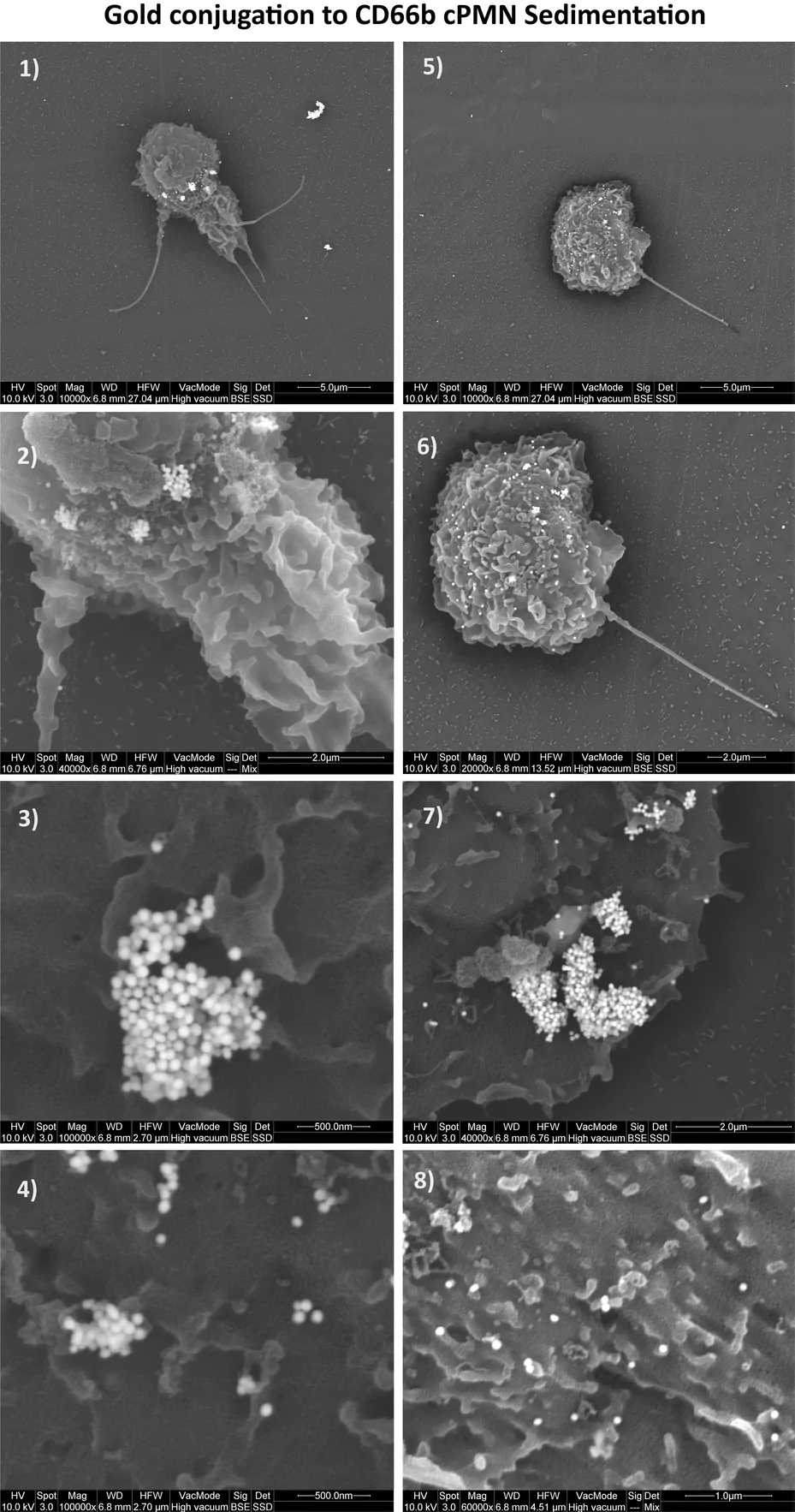
Visualization of CD66b distribution on cPMNs after isolation by sedimentation only. Images (1–4) and (5–8) each represent a single PMN cell at a different magnification (visible in backscatter electron microscopic image as white dots due to higher atomic number: gold particles coupled to anti‐CD66b antibodies). CD66b distributed at several sites across the cell surface.

**Figure 6:**
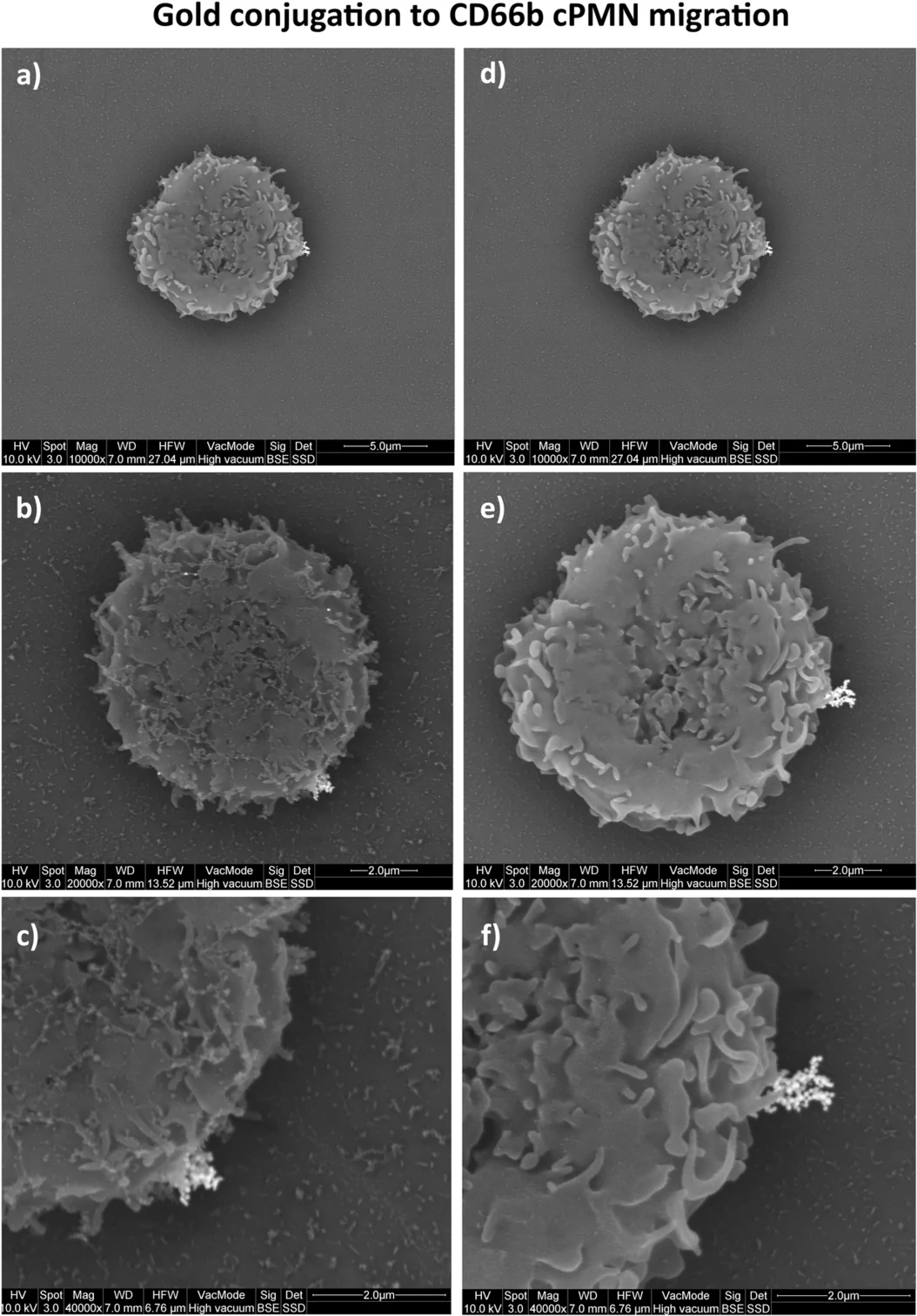
Visualization of CD66b distribution of isolated cPMNs that migrated through collagen gel. Images (a–c) and (d–f) each represent a single PMN cell at different magnifications (visible in backscatter electron microscopic image as white dots due to higher atomic number: gold particles coupled to anti‐CD66b antibodies). A concentrated accumulation in one place can be spotted.

**Figure 7:**
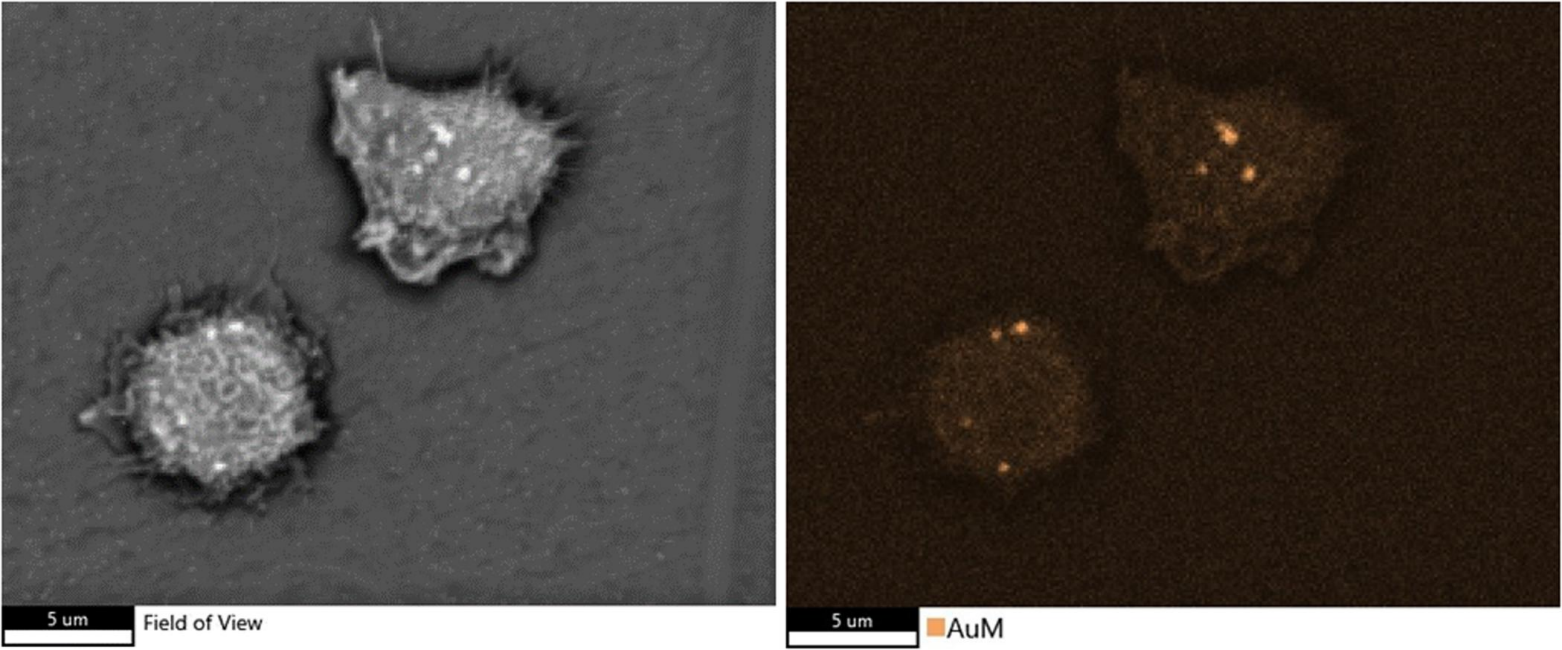
Exemplary EDX‐mapping to confirm the presence of elemental gold in the area of clustered surface epitopes on the cell surface. Left image: backscatter electron image for area selection; right image: pixel intensity reflects the presence of elemental gold (orange) only on the cell surface. For these SEM images, cPMN sedimentation samples were used.

#### 3.2.5 Oral PMNs Showed No Chemotactic Migration

Stimulation with fMLP caused cPMNs to migrate into and through the type I collagen matrix along the fMLP chemotactic gradient. The median track length was 84 µm, as determined from *n* = 6261 tracks observed over a period of 30 min after 5 h runtime. In contrast, oPMNs neither migrated into the gel nor moved when introduced directly into it.

## 4 Discussion

### 4.1 Expression of LOX‐1 on PMNs in Peripheral Blood and Saliva

The purpose of this study was to investigate the numbers, surface epitopes, migratory properties, and morphology of oral and cPMNs in healthy volunteers. PMNs are required for homeostatic immunity and contribute to clinically healthy periodontal tissue. In the long term, however, overwhelming PMN immune activity can contribute to structural damage and chronic inflammation as observed in periodontal diseases. The balance between initiating the healing process and destruction is delicate and heavily depends on how acute inflammation progresses to chronic inflammation [35]. During transmigration from circulation to the oral cavity, PMNs are exposed to new environments, resulting in activation and a subsequent change in their phenotype and function [9].

The results of this study showed a significant higher expression of LOX‐1 on oPMNs than on cPMNs (see Figure 1). Under physiological conditions, PMNs can leave the blood circulation, enter the gingival crevices and are “transported” by gingival crevicular fluid to mix with saliva [11]. They seem to change their activation state when they either arrive in or are on their way to the oral cavity, as expressed by their changed surface epitope LOX‐1. Therefore, LOX‐1 seems to be important for the maintenance of physiological PMN activity in the oral cavity. Interestingly, the scavenger receptor LOX‐1 is primarily known for promoting endothelial dysfunction. This occurs through the uptake of oxidized and electronegative LDL in ECs, which induces proatherogenic signaling and plaque formation [14]. However, LOX‐1 is present on many cell types including macrophages, lymphocytes and PMNs, thus highlighting the importance of this receptor in various aspects of inflammatory processes, including atherosclerotic plaque formation [14, 16, 17].

Although LOX‐1 is primarily expressed on ECs, collectively, these receptors seem to work as pattern recognition proteins as part of the innate immune system (presence on PMN surfaces). LOX‐1 guard against extrinsic microorganisms, hazardous materials, intrinsic damage and senescence‐related substances [14, 36]. Moreover, LOX‐1 works as an adhesion molecule for negatively charged phospholipids on platelets on ECs, enhancing the release of endothelin‐1 from ECs promoting endothelial dysfunction [37, 38]. Together with these properties, LOX‐1 works as an adhesion molecule, not only for activated platelets but also for PMNs [15].

Intriguingly, LOX‐1 is involved in a plethora of processes relevant to the pathogenesis of certain malignancies and plays a causative role in tumor initiation and progression via angiogenesis‐dependent mechanisms [14]. In this context, LOX‐1 induced PMN migration and recruitment to high‐metastatic tumor via CCL2 secretion from tumor ECs [39]. Tumor‐suppressive PMNs can be identified using LOX‐1 [40]. LOX‐1 is also the sole marker of granulocytic myeloid‐derived suppressor cells (MDSC) measurable in whole blood [14, 17, 41]. In oral squamous cell carcinoma, endoplasmic reticulum‐stressed PMNs exhibited immunosuppressive activity characterized by increased LOX‐1 expression [42]. Our findings that LOX‐1 is significantly increased on oPMNs, even in healthy individuals, may contribute to a better understanding of the homeostasis of the oral cavity in both healthy and malignant conditions.

Nevertheless, in the absence of malignancy, Sakurai and Sawamura described LOX‐1 expressed on ECs as an adhesion molecule for activated PMNs. According to Zhang and colleagues, LOX‐1 seems to be important for PMN recruitment to the sites of infection [15, 43]. Both observations suggest that the increased expression of LOX‐1 in oPMNs may be due to physiological recruitment through the endothelium during diapedesis, rather than from contact with bacteria in saliva.

### 4.2 Stimulation of LOX‐1 on PMNs in Peripheral Blood and Saliva

CPMNs can be activated by TNF‐α and PMA in a way that they upregulate LOX‐1 on their surfaces [36, 44]. Regardless of the activation time, oPMNs expressed significantly more LOX‐1 antigens on their surfaces than cPMNs. OPMNs seem to have already transported most of the stored intracellular LOX‐1 to the cell surface. As a consequence, oPMNs cannot be stimulated by TNF‐α or PMA with regard to LOX‐1 expression. In PMNs of saliva, LOX‐1 expression remains on a consistently high level and cannot be stimulated further.

One possible explanation for the high level of LOX‐1 may be that oPMNs have already adapted to the high antigen exposure by the permanent abundance of bacteria found in the oral cavity, resulting in a state of permanent activation [45]. The lack of a permanent bacterial presence in peripheral blood means that cPMNs can still be stimulated. In line with our results and the hypothesis that oPMNs are in a different state of activation, Rijkschroeff and colleagues postulated that oPMNs are in a more mature stage of their life cycle than cPMNs because more late‐stage apoptotic/necrotic cells were found among oPMNs [46]. Thereby, expression of LOX‐1 on PMN surfaces seems to play an important role in the regulation of PMN apoptosis because Li and colleagues observed decreased PMN apoptosis by LOX‐1 inhibition [47].

### 4.3 Expression and Stimulation of CD11b, CD62L, CD66b and Oxidative Burst on PMNs in Peripheral Blood and Saliva

Gingival crevices are suggested to be the main point of entry for oPMNs into the oral cavity from the bloodstream, whereas only a small proportion of cells originate from salivary glands [9]. In our experiments, we found a significantly higher expression of CD11b in cPMNs than in oPMNs, regardless of the state of activation. OPMNs have already migrated through the blood vessels, therefore, it seems appropriate that CD11b, which is responsible for adherence to the vascular endothelium, is expressed less. Regardless of the state of activation, CD66b (mediator of PMN phagocytosis) showed no significant difference between cPMNs and oPMNs [13]. This lack of difference suggests that phagocytosis is an important defense mechanism that is ready to be used anywhere regardless of where the PMNs are located. However, the results of CD11b and CD66b expression in our study are in contrast to those found by Nicu and colleagues who described a higher expression of CD11b and CD66b on oPMNs than on cPMNs. Differences in PMN isolation processes (noncentrifugation in our study vs. centrifugation used by Nicu and colleagues) may explain the observed variations, as centrifugation can impair PMN surface receptor assays [11, 48].

Without stimulation, there was no difference in CD62L (essential for transmigration through blood vessels) between oPMNs and cPMNs. Only when stimulated with PMA the level of CD62L decreased over time in cPMNs. Once diapedesis has been completed and PMNs have arrived in the oral cavity, CD62L seems to have served its purpose, as further stimulation does not lead to additional CD62L downregulation [2].

In our experiments, no PMNs could be detected by flow cytometry after 120 min of PMA activation, probably because, at this time, the PMNs demonstrated NETosis due to PMA activation, meaning no PMN‐attributable fluorescence could be generated. Furthermore, without activation, oPMNs produced significantly more ROS than cPMNs. ROS production could only be increased in cPMNs by fMLP and PMA, even beyond the level of oPMNs.

At this point, it should be mentioned that the intrinsic ROS production measured by FACS exclusively reflects the ROS production from viable cells, as the gating strategy effectively filtered out all nonvital cells. The proportion of nonvital cells was significantly higher in the oPMNs, and it is strongly assumed that these nonvital cells do not exhibit ROS production. In the ROS production measurements, nonvital cells were not captured, as living cells were identified by plotting the SSC against the distinct fluorescence channel, thereby excluding nonvital cells from the analysis [4].

This result further supports the above findings regarding LOX‐1 expression that oPMNs already seem to be in a more active state, meaning that they cannot be activated further. cPMNs, on the other hand, can be activated. Similar results were found in earlier publications by Rijkschroeff and colleagues who also observed that, without any additional stimulation, the oPMNs of healthy subjects were physiologically more activated than cPMNs, as indicated by the higher expression of CD11b and CD66b, lower CD62L and higher constitutive ROS levels. In addition, oPMNs released comparable ROS levels as cPMNs in response to bacterial stimulation, which indicates that oPMNs are already activated by the surrounding bacteria and further activation is not possible. However, that previous research was conducted without considering LOX‐1. Nonetheless, it is crucial to acknowledge LOX‐1 as an integral component necessary for future research to enhance our understanding of the fundamental processes involved in the oral cavity [6, 46].

To sum up, oPMN upregulate molecules that reflect their activation in the oral cavity [9]. We agree that oPMNs seem to be at a later stage in their life cycle than cPMNs. Nevertheless, they are still capable of performing innate immune functions, which suggests that they have the potential to contribute to a healthy oral ecosystem [46].

### 4.4 Scanning Electron Microscopy: Different PMN Surface Morphology

Differences in surface characteristics, which had already been determined using flow cytometry, were also visualized using SEM. While cPMNs showed a smoother surface with fewer protrusions after migration, oPMN surface appeared porous (see Figure 4).

In our study, the expression of CD66b on the surface of PMNs was demonstrated descriptively for the first time, using specific gold‐labeled antibodies on the surface. Labeling CD66b with antibodies coupled to gold beads confirmed that the observed objects were indeed PMNs. On the other hand, they also allowed conclusions to be drawn about the distribution of the surface epitope CD66b of cPMNs. CD66b was found bundled in single clusters on cPMNs. No gold from gold‐coupled antibodies was visible in the supernatant or saliva alone. After the migration through collagen gel, the lamellae become fewer and the surface smoother. Only clusters for CD66b were left showing accumulation at one site (see Figures 5 and 6). CD66b appears to be downregulated or at least bundled after chemotactic migration along an fMLP gradient through collagen gel (comparable to flow cytometric results after activation with fMLP, see Section 3.2.5, 5).

The appearance of the oPMNs is similar to that of an image published by Giaglis and colleagues, in which PMNs were activated with PMA for 3 h [24]. Based on the hypothesis that PMN activation alters the PMN surface, it could therefore be concluded that oPMNs were also activated in the same way as PMNs when incubated for 3 h with PMA as performed by Giaglis and colleagues. This hypothesis is supported by the lack of activation of oPMNs by PMA and the loss of stimulatory capacity in the chemotaxis model (see Sections 3.2.2 and 3.2.5). The state of activation of PMNs can therefore not only be determined by different expression levels of surface epitopes in flow cytometry, but may also be reflected morphologically in the appearance of PMNs at high magnification [24].

### 4.5 oPMNs Lose Stimulatory Capacity in the Chemotaxis Model

Since oPMNs showed no migration upon chemotactic attraction with fMLP in our experiments, high LOX‐1 expression appears to be associated with impaired chemotaxis and chemokinesis. In our model, PMNs needed to move through the reservoirs first until they could be observed in the 3D‐µ‐slide channel. Since we could not see oPMN migration in the channel in our experiments, we embedded PMNs directly into the gel in an additional series of experiments. However, despite this modification, oPMNs did not migrate. Therefore, we concluded that oPMNs lost their chemotactic stimulatory capacity. Our results coincide with the results found by Domnich and colleagues who observed a significant decrease in PMN migratory capacity once PMNs have transmigrated from the bloodstream to the oral cavity. In addition, they found a reduced fMLP receptor in oPMNs, which may explain the lower stimulability in our chemotaxis model [9]. On closer examination, it is not surprising that oPMNs do not respond to fMLP the same way as cPMN due to the prior exposure to bacteria of oPMN. Such exposure can lead to the desensitization of the fMLP receptor, and these receptors become less responsive after repeated stimulation [49]. Thereby, the chemokine receptor 1 (CCR 1) appears to be primarily responsible for the sensitization effect, and the desensitization of bacterial activation is dependent upon receptor phosphorylation [49, 50].

Due to the nature of our live‐cell‐imaging assay, a quantitative chemotaxis analysis was not possible in the absence of cell migration, as no measurable parameters could be obtained when chemotaxis is entirely lacking. In our experiments, LOX‐1–expressing cells showed no movement at all, which meant that only a qualitative assessment could be made, indicating a complete loss of chemotactic capacity. However, this observation is in line with previous reports: Al‐Banna and Lehmann demonstrated that LOX‐1 deletion in mice enhances neutrophil chemotaxis to infection sites in CLP‐induced sepsis [51]. Wu and colleagues took it further and reported that LOX‐1 signaling itself is involved in impaired neutrophil chemotaxis during sepsis [52].

However, PMNs also show impaired immune functions in inflammatory processes in the oral cavity. Almost 50 years ago, Cianciola and colleagues described that PMNs from patients with periodontitis have impaired migration and phagocytic function [53]. It has been hypothesized that in the absence of scavenger molecules, bactericidal species (ROS and enzymes) from PMNs may harm host tissues. In a scenario of a periodontal pocket, in which PMNs encounter an overwhelming biofilm, host‐derived cells may die and, in a final attempt to destroy the biofilm, turn their lethal weaponry inside out via extracellular degranulation, leading to collateral damage. ROS in interaction with biofilm are considered to provoke periodontal tissue destruction [45]. Therefore, investigating oPMN function remains an important research topic to better understand the phenomena of resistance and resilience toward biofilm‐induced gingival inflammation in the oral cavity [54]. In this instance, particularly the transition between the healthy state and gingivitis is known to be a very individual process, as shown by many experimental gingivitis studies [54, 55, 56, 57].

Overall, the observation that oPMNs intrinsically produce a significant amount of ROS even in the absence of artificial external stimulation (such as PMA) suggests that they are in a more activated state. Intrinsic oPMN activation may be due to bacteria in the saliva that are actively engaged in phagocytosing. This activation is particularly interesting when compared to cPMNs, which typically require stimulation to produce ROS. The high bacterial load in saliva may lead to a constant state of activation for oPMNs, enabling them to respond quickly to microbial threats [7]. Such differences in behavior between oPMNs and cPMNs highlight the unique challenges faced by immune cells in the oral cavity, yet it seems to be an area that has not received much attention in the literature so far. Further exploration of this phenomenon could provide valuable insights into the immune response in the oral cavity and its implications for oral health.

Overall, further research is needed to determine which microbiological, immunological, and biochemical biomarkers would be useful in future investigations of the resistance and resilience in the oral cavity and excessive PMN activation [54].

### 4.6 Characteristics and Limitations

The sample size of the present study represents a limitation. However, this study serves as a pilot study with the aim of identifying new correlations and phenomena. Additionally, Casy cell count measurements were primarily conducted as a preliminary assessment to provide an initial estimate of cell numbers. The facts are described for the first time, whereby the examination using an SEM was used exclusively for descriptive purposes. As mentioned in the introduction, when PMNs migrate from the circulatory system to the oral cavity, they gain specific phenotypic and functional markers. Thereby, the selected surface epitopes (CD11b, CD62L, and CD66b) are essential for PMNs to assume and fulfill their functions. In consideration of their pivotal role in PMN function, these epitopes were selected for analysis [5, 13].

Our study differs from earlier work in a crucial methodological aspect. We used a refined PMN isolation technique that avoids centrifugation‐induced activation, which has been shown in our recent publications [1, 2] to significantly influence neutrophil surface marker expression. Most of the previously cited studies employed either centrifugation or erythrocyte lysis, and both of these methods can introduce artefacts. Thus, our approach allowed a more physiological assessment of native and unactivated oPMNs and cPMNs, providing added value even when analyzing previously described markers.

Nevertheless, our method had limitations due to conventional flow cytometry technology. Our flow cytometry analyses were performed using a BD FACSCalibur system, which represents a legacy system of cytometers. Adjustments in panel design and data acquisition were required, particularly regarding the number of available fluorescence channels and the gating strategy. Future studies should aim to refine methodology, which would allow the inclusion of a greater number of markers within a single panel and help overcome current technical limitations.

Despite the limitations regarding technology and sample size, we observed interesting results, such as LOX‐1 expression on PMN surface, which warrant further investigation. Our findings serve as a basis for further studies, which would not have been feasible without the insights gained from the current research.

## 5 Conclusion

To the best of our knowledge, this is the first report of the high physiological expression of LOX‐1 on PMNs in the oral cavity. LOX‐1 seems to be a marker for PMNs in a more activated state (possibly due to continuous stimulation by the oral microbiota) without any substantial additional activation capacity.

## Author Contributions

Richard F. Kraus codeveloped the methodology, performed advanced data analyses, and wrote the original draft of the manuscript. Vanessa J. Stampfer conducted the flow cytometry experiments and performed the associated data analyses. Konstantin J. Scholz led and validated the electron microscopy investigations and drafted the corresponding section of the manuscript. Michael A. Gruber conceived the study concept, developed and refined the methodology, contributed to data interpretation, and validated the results. Fabian Cieplik conceived the study, carried out data evaluation, and provided overall supervision. All authors contributed to the writing of the manuscript, critically revised the content, and proofread.

## Funding

The authors received no specific funding for this work.

## Ethics Statement

This study was approved by the Internal Review Board of the University of Regensburg (Reference 22‐3062‐101).

## Consent

Written informed consent was obtained from all volunteers included in this study before blood sampling.

## Conflicts of Interest

The authors declare no conflicts of interest.

## Supporting information


**Figure S1:** Light microscopic images of saliva sample preparation and mucin separation by Filtration.

**Figure S2:** The first gating strategy involved running the samples via front‐side‐scatter (FSC‐H) and side scatter (SSC‐H), followed by staining with CD66b (specific for neutrophil granulocytes), and concluding with division into CD62L and CD11b. Abbreviations: Granulocytes = PMNs; SSC‐H = side scatter height; FSC‐H = forward scatter Height.

**Figure S3:** The second gating strategy also used FSC + SSC, followed by staining with CD15‐FITC and then LOX‐1‐APC. Abbreviations: Granulocytes = PMNs; SSC‐H = side scatter height; FSC‐H = forward scatter Height.

**Figure S4:** The third gating strategy was for the respiratory burst. Dead cells were stained using 10 μL of propidium iodide (PI). Only PI‐negative (living) cells were included in subsequent analyses to assess their ROS production. Abbreviations: Granulocytes = PMNs; SSC‐H = side scatter height; FSC‐H = forward scatter Height.

Supporting Information

Supporting Information

**Table 1:** Proportion of dead cells (determined by staining with propidium iodide) subdivided into cPMN and oPMN and activation. Data is shown as Median with (Range) in brackets.

## Data Availability

Data are available upon reasonable request from the corresponding author due to privacy/ethical restrictions.
