# Supplementary material for: Differences in Lectin‐Like Oxidized Low‐Density Lipoprotein Receptor‐1 Expression and Chemotaxis Between Neutrophils in the Oral Cavity and Those in Whole Blood
Source: Immun Inflamm Dis. 2026 Mar 18;14(3):e70390. doi: 10.1002/iid3.70390 (PMC13757336; doi:10.1002/iid3.70390)
Supplement: Supplementary file 1 [file IID3-14-e70390-s001.docx]

Table 2: Information on post-hoc tests used

The following file contains the post-hoc tests used for multiple comparisons in the event of significant differences:

1) ANOVA (for normal distribution): Dunnet-T3 or Bonferroni
2) Kruskal-Wallis test for non-normal distribution: Dunn-Bonferroni correction.

# Expression of LOX-1 (Fluorescence of LOX-1 [MFI])

| Illustration | Incubation time | Used Post-hoc test |
| --- | --- | --- |
| Illustrated in Figure 1a) | 20 min | Dunnet-T3 |
| Illustrated in Figure 1a) | 40 min | Dunnet-T3 |
| Illustrated in Figure 1a) | 60 min | Bonferroni |
| Illustrated in Figure 1b) | 20 – 120 min | Dunnet-T3 |

# Expression of CD62L (Fluorescence of CD62L [MFI])

| Illustration | Incubation time | Used Post-hoc test |
| --- | --- | --- |
| Illustrated in Figure 1c) | 20 min | Dunnet-T3 |
| Illustrated in Figure 1c) | 60 min | Bonferroni |
| Illustrated in Figure 1d) | 20 – 120 min | Dunnet-T3 |

# Expression of CD11b (Fluorescence of CD11b [MFI])

| Illustration | Incubation time | Used Post-hoc test |
| --- | --- | --- |
| Illustrated in Figure 2b) | 20 – 120 min | Dunnet-T3 |

# Expression of CD66b (Fluorescence of CD66b [MFI])

| Illustration | Incubation time | Used Post-hoc test |
| --- | --- | --- |
| Illustrated in Figure 2c) | 20 min | Bonferroni |
| Illustrated in Figure 2c) | 40 min | Dunnet-T3 |
| Illustrated in Figure 2d) | 20 – 120 min | Dunnet-T3 |

# Oxidative Burst (Fluorescence of Rhodamine 123 [MFI])

| Illustration | Incubation time | Used Post-hoc test |
| --- | --- | --- |
| Illustrated in Figure 3a) | 20 – 120 min | Dunn-Bonferroni |
