# Supplementary material for: Differences in Lectin‐Like Oxidized Low‐Density Lipoprotein Receptor‐1 Expression and Chemotaxis Between Neutrophils in the Oral Cavity and Those in Whole Blood
Source: Immun Inflamm Dis. 2026 Mar 18;14(3):e70390. doi: 10.1002/iid3.70390 (PMC13757336; doi:10.1002/iid3.70390)
Supplement: Supplementary file 2 [file IID3-14-e70390-s004.docx]

Table 1: Proportion of dead cells (determined by staining with propidium iodide) subdivided into cPMN and oPMN and activation. Data is shown as Median with (Range) in brackets.

|  | Activation | Median (Range) [%] |
| --- | --- | --- |
| cPMN | Without | 0.18 (10.7) |
|  | fMLP | 0.23 (1.5) |
|  | PMA | 0.37 (1.9) |
| oPMN | Without | 17.1 (38.9) |
|  | fMLP | 12.9 (27.7) |
|  | PMA | 29.4 (42.1) |
