# Supplementary material for: Differences in Lectin‐Like Oxidized Low‐Density Lipoprotein Receptor‐1 Expression and Chemotaxis Between Neutrophils in the Oral Cavity and Those in Whole Blood
Source: Immun Inflamm Dis. 2026 Mar 18;14(3):e70390. doi: 10.1002/iid3.70390 (PMC13757336; doi:10.1002/iid3.70390)
Supplement: Supplementary file 3 [file IID3-14-e70390-s005.docx]

# Descriptions for figures in the supplement

**Figure S1:**	Light microscopic images of saliva sample preparation and mucin separation by

Filtration

**Figure S2:**	The first gating strategy involved running the samples via front-side-scatter (FSC-H) and side scatter (SSC-H), followed by staining with CD66b (specific for neutrophil granulocytes), and concluding with division into CD62L and CD11b. *Abbreviations: Granulocytes = PMNs; SSC-H = side scatter height; FSC-H = forward scatter Height*

**Figure S3:**	The second gating strategy also used FSC + SSC, followed by staining with CD15-FITC and then LOX-1-APC. *Abbreviations: Granulocytes = PMNs; SSC-H = side scatter height; FSC-H = forward scatter Height*

**Figure S4:**	The third gating strategy was for the respiratory burst. Dead cells were stained using 10 μL of propidium iodide (PI). Only PI-negative (living) cells were included in subsequent analyses to assess their ROS production. *Abbreviations: Granulocytes = PMNs; SSC-H = side scatter height; FSC-H = forward scatter Height*
